# Malnutrition and Insulin Resistance May Interact with Metabolic Syndrome in Prevalent Hemodialysis Patients

**DOI:** 10.3390/jcm12062239

**Published:** 2023-03-14

**Authors:** Shuzo Kobayashi, Yasuhiro Mochida, Kunihiro Ishioka, Machiko Oka, Kyoko Maesato, Hidekazu Moriya, Sumi Hidaka, Takayasu Ohtake

**Affiliations:** 1 Kidney Disease and Transplant Center, Shonan Kamakura General Hospital, Kamakura 247-8533, Japan; 2 Nephrology, Tokyo Nishi Tokushukai Hospital, Akishima 196-0003, Japan

**Keywords:** hemodialysis, metabolic syndrome, abdominal obesity, inflammation, atherosclerosis

## Abstract

Background: We sought to determine the prevalence of metabolic syndrome (Mets) and whether 100 cm^2^ of visceral fatty area (VFA) measured by computed tomography (CT) validates the criteria of waist circumference (WC) in hemodialysis (HD) patients. Methods: The study comprised 141 HD patients. Mets was defined according to the criteria of Adult Treatment Panel III (ATP III) and the modified criteria of National Cholesterol Education Program (NCEP) that defines abdominal obesity as a WC of >=85 cm in men and >=90 cm in women. Results: The prevalence of Mets was 31.9% in men and 13.6% in women. However, the prevalence of patients with a body mass index over 25 in all HD patients was only 11.2%. The visceral fatty area (VFA) measured by CT showed a strong positive correlation with WC. The patients with Mets, comparing with those without Mets, have significantly shorter duration of HD, higher high-sensitive C-reactive protein, and higher Homeostatic Model Assessment for Insulin Resistance (HOMA-IR). In the patients with Mets, there was a significant negative correlation between HOMA-IR and serum albumin levels. Multivariate logistic regression analysis showed that HOMA-IR and short duration of HD were chosen as independent risk factors for Mets. Conclusions: Mets is more prevalent in HD patients. In Japanese HD patients, 100 cm^2^ of VFA corresponded to a WC of 85 cm in men and 90 cm in women, thus confirming the validity of the modified criteria. HOMA-IR and serum albumin were significantly correlated in HD patients with Mets.

## 1. Introduction

In patients on hemodialysis (HD), malnutrition and its related inflammation are known to cause atherosclerosis (malnutrition, inflammation, atherosclerotic syndrome; MIA syndrome) [1], thus leading to cardiovascular disease (CVD). Likewise, metabolic syndrome (Mets), characterized by abdominal obesity, hypertriglyceridemia, low high-density lipoprotein (HDL) cholesterol level, high blood pressure, and high fasting glucose level [2], is also known to be a major leading cause of CVD in the general population [3]. Mets has been associated with an increased risk for diabetes mellitus and CVD, as well as increased CVD and all-cause mortality [3,4,5]. Chen et al. reported that Mets is prevalent and might be an important factor in the cause of chronic kidney disease (CKD) [6]. In Japan, Mets is a significant determinant of CKD in men under 60 years of age [7].

Besides the fact that Mets is one of the risk factors in CKD, it is important to note that we need to know the prevalence of Mets and its associated factors in maintenance HD patients because malnutrition develops with longer HD duration. In this regard, little information is available, although at the initiation of renal replacement therapy (RRT), there is a report showing that Mets is highly prevalent in incident dialysis patients [8]. Unfortunately, body mass index (BMI) is used instead of the criteria of waist circumferences (WC), and no data on fasting blood are available in that report. Moreover, information on associated factors is not provided.

The pathogenesis of Mets and the relationship between Mets and CVD lie in insulin resistance [9]. Insulin resistance is known to develop at an early stage of non-diabetic CKD [10]. We reported a similar result using a hyperinsulinemic euglycemic glucose clamp method and also showed that acidemia and dyslipidemia are independently associated with insulin resistance in CKD [11]. Although RRT improves insulin resistance [12], insulin resistance is still occasionally found in maintenance HD patients [13]. However, it remains unknown concerning the relationship between Mets and insulin resistance in hemodialysis patients.

Therefore, in the present study using fasting blood samples, we first assessed the prevalence of Mets in maintenance HD patients according to the criteria of the Adult Treatment Panel III (ATP III) [2] using modified criteria of WC. We studied the associated factors including insulin resistance expressed by the Homeostasis Model Assessment of Insulin Resistance (HOMA-IR) [14]. Finally, using computer tomography (CT) [15], we confirmed whether or not visceral fat area (VFA) greater than 100 cm^2^ corresponds to a WC of 85 cm in men and 90 cm in women, respectively.

## 2. Materials and Methods

### 2.1. Study Design and the Subjects

The present study was cross-sectional observational study conducted in our hospital. The protocol was approved by the Tokushukai Group Institutional Review Board (TGE1897-024) and adhered to the tenets of Declaration of Helsinki. The potential subjects comprised 162 patients who were on maintenance HD therapy in dialysis center in our hospital in December 2005. The end date of patient recruitment was 31 December 2005. Patients aged 20 years old or more were enrolled, and there was no upper restriction in age for study entry. Patients with insulin treatment were essentially excluded in this study. Data were collected from maintenance HD patients in December 2005 unless they had acute illness (9 patients) or post-operative conditions (4 patients) within 3 months prior to this study. The patients within 3 months of the initiation of HD (8 patients) were also excluded. The patients visited to our hospital for a 1-day annual check of complications.

The study comprised 141 HD patients (97 men, 44 women). These patients recruited in the present study corresponded to 90% (141/162 patients) of all patients in our hospital and had been treated by regular dialysis for more than 3 months.

### 2.2. Blood Sampling, WC, and VFA

Blood was drawn in the morning after an overnight fast of at least 12 h on non-dialysis day in the middle of week. EDTA-plasma was used for glucose, insulin, and lipids, and serum for other biochemical assays. Glucose was measured by a glucose oxidase method. Insulin was measured by radioimmunoassay (Insulin RIA-BEAD II: Dinabot Co., Tokyo, Japan). Total cholesterol (TC) and triglycerides (TG) were measured enzymatically. HDL cholesterol was measured after precipitating apolipoprotein B-containing lipoproteins with dextran sulfate and magnesium chloride. High-sensitive C-reactive protein (hsCRP) was measured using a nephelometric immunoassay. WCs of the patients were also measured at a standing position. Finally, 92 patients in men and 20 patients in women who agreed to undergo CT examination received CT examination for measuring VFA, respectively.

### 2.3. Assessment of Insulin Resistance Using HOMA-IR

Insulin resistance was assessed using HOMA-IR originally described by Mathew et al. [14]. HOMA-IR was calculated using the following formula: HOMA-IR = fasting glucose (mmol/L) × fasting insulin (μU/mL)/22.5.

### 2.4. BMI and Measurement of VFA Using CT

BMI was calculated as the weight in kilograms divided by the height in meters squared.

The amount of abdominal and visceral fat deposition was assessed by CT. The area of the subcutaneous fat and visceral fat was measured in a single cross-sectional scan at the level of the umbilicus. An image histogram was computed for the subcutaneous fat layers in order to determine the range of CT numbers for the fat tissue. The total fat area was then calculated by counting the pixels that had intensities within the selected range of CT numbers. The intraperitoneal space was defined by tracing its contour on the scan image. The total area with the same CT numbers was considered to represent VFA [15].

### 2.5. Mets Criteria

The ATP III [2] report defines Mets as a constellation of risk factors of metabolic origin including increased abdominal obesity, high triglyceride, low HDL cholesterol, elevated blood pressure, and elevated fasting blood glucose. Elevated blood pressure was defined as systolic or diastolic blood pressure of 130/85 mmHg or higher; low HDL cholesterol level was defined as less than 40 mg/dL; high serum TG levels were defined as 150 mg/dL or more; and elevated fasting glucose level was defined as 110 mg/dL or more. Finally, WC of 85 cm or more in men and 90 cm or more in women was defined as abdominal obesity in Japan as modified NCEP criteria [7].

### 2.6. Measurement of Blood Pressure

Blood pressure (BP) was measured with a standard mercury sphygmomanometer and cuffs adapted to arm circumferences after the patients had rested in the supine position for at least 5 min prior to HD on the first HD session of a week. Hypertension was also defined as the use of one or more antihypertensive drugs.

### 2.7. Statistical Analysis

Continuous variables were expressed as mean ± standard deviation (SD) when normally distributed or as median (interquartile range [IQR]) when non-normally distributed. Skewed variables underwent log transformation before statistical analysis. The prevalence of Mets and its individual components (elevated BP level, high plasma glucose level, high triglyceride level, low HDL cholesterol level, and abdominal obesity), as well as the number of Mets components (0, 1, 2, 3, 4, or 5), was determined for the overall study sample. Univariate or multivariate logistic regression analysis was also applied for the determinants of Mets. A *p*-value of less than 0.05 was considered statistically significant. These were analyzed using statistical software (StatView 5; SAS Institute Inc., Carry, NC, USA) for Windows personal computer.

## 3. Results

The mean age was 67 years, with a range of 34–89 years. Table 1 summarizes the baseline characteristics of the subjects. Body weight, body height, rate of current smoker, rate of diabetes mellitus, TC, and HDL-C were significantly different between men and women, as shown in Table 1.

The prevalence of Mets was 31.9% in men, 13.6% in women, and 26.2% in total according to the modified criteria of NCEP using a definition of a WC of 85 cm or more in men and 90 cm or more in women. The prevalence of HD patients with each Mets component in men and women was shown in Table 2. The prevalence of a WC of 85 cm or more in men and 90 cm or more in women was 53.6% and 25%, respectively. The number of Mets components present in men was 3.8% with no Mets risk factors, 36.5% with one, 44.2% with two, 7.7% with three, and 7.7% with four. The number of Mets components present in women was 0% in no Mets risk factors, 45.4% with one, 54.5% with two, and 0% with three and/or four.

**Figure 1:**
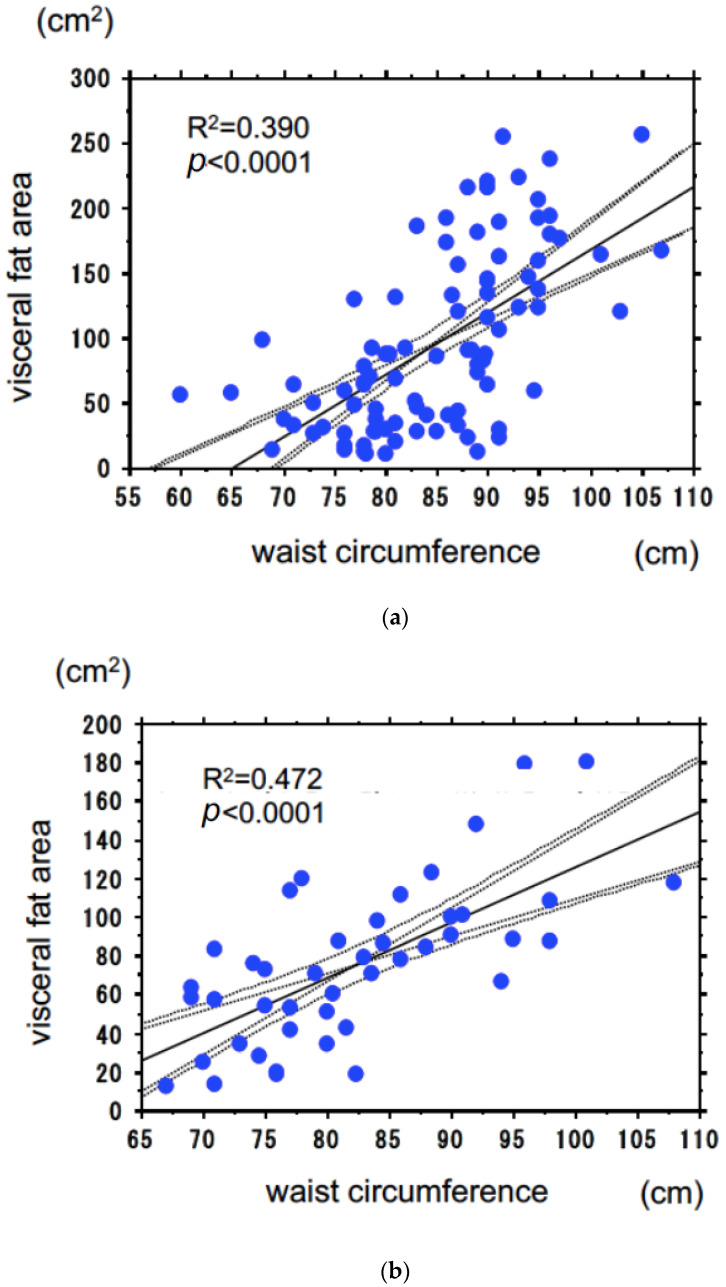
The relationship between visceral fat area and waist circumferences in men (**a**), the relationship between visceral fat area and waist circumferences in women. (**b**). Abbreviations: VFA visceral fat area, WC waist circumference.

The VFA measured by CT showed a strong positive correlation with WC in both men (R^2^ = 0.390, *p* < 0.0001) and women (R^2^ = 0.472, *p* < 0.0001) as shown in Figure 1. In Japanese HD patients, 100 cm^2^ of VFA corresponded to a WC of 85 cm in men and 90 cm in women, thus confirming the validity of the modified criteria.

HOMA-IR showed a skewed distribution with a median of 0.922, and 20% of the patients had the value greater than 2.0 of HOMA-IR.

The patients with Mets, comparing with those without Mets, had significantly greater WC, shorter duration of HD, greater BMI, higher hsCRP, higher HOMA-IR, higher FBS, and higher TG, as shown in Table 3. In patients undergoing HD for more than 10 years, the prevalence of Mets became 10% (3 patients/30 patients) (data not shown).

**Figure 2:**
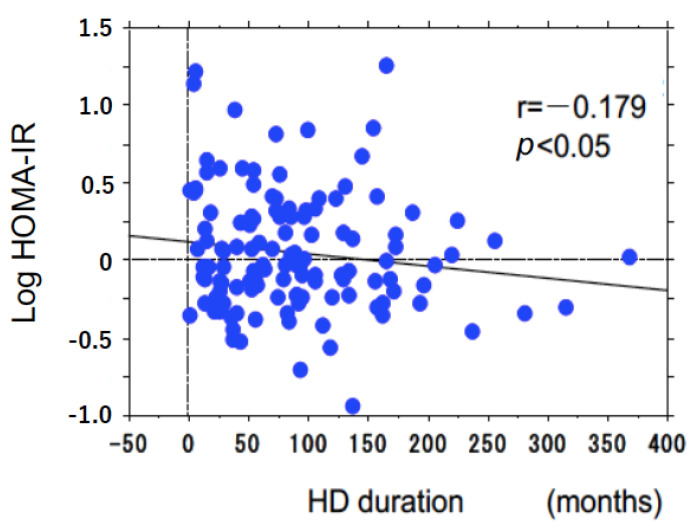
The relationship between HOMA-IR and HD duration (months) (*p* < 0.05, r = −0.179). Abbreviations: HOMA-IR Homeostasis Model Assessment of Insulin resistance, HD hemodialysis.

In all patients, a significant negative correlation between serum albumin and hsCRP (R^2^ = 0.101, *p* < 0.001), and a negative weak correlation between the duration of HD and HOMA-IR (R^2^ = 0.032, *p* < 0.05) were found [Figure 2]. On the contrary, the prevalence of serum albumin levels less than 3.7 g/dL was 45.2% in men and 70.5% in women, respectively. The prevalence of the patients with BMI less than 18.5 was 17.5% in men, and 27.3% in women, respectively. The prevalence of patients with a BMI greater than 25 in all HD patients was only 11.2%.

**Figure 3:**
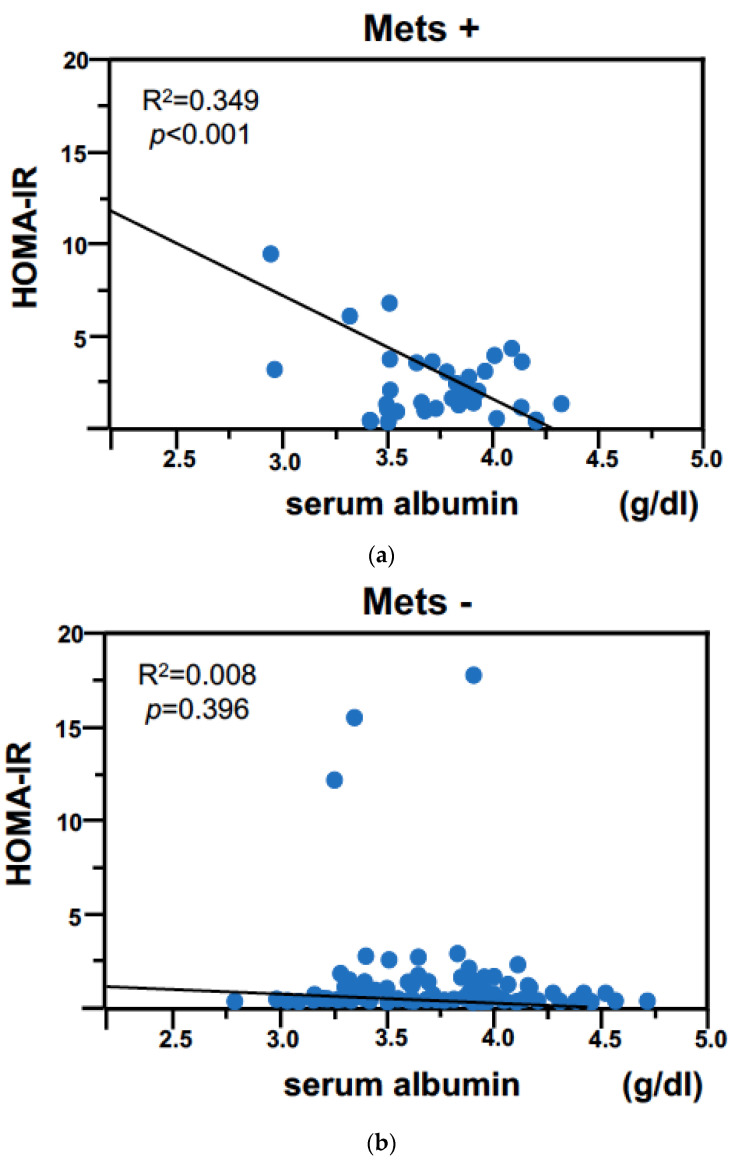
The relationship between HOMA-IR and serum albumin in patients with Mets (**a**) and relationship between HOMA-IR and serum albumin in patients without Mets (**b**).

Regarding the correlation between HOMA-IR and serum albumin levels, there was a significant negative correlation only in patients with Mets (R^2^ = 0.349, *p* < 0.001), while in patients without Mets, there was no significant correlation (R^2^ < 0.001, *p* = 0.957) (Figure 3). In a study of univariate regression analysis associated with HOMA-IR, only serum albumin level was chosen as a significant determinant [Table 4] in patients with Mets, while other parameters, including age, HD duration, WC, BMI, hsCRP, TC, TG, and HDL cholesterol, did not show any significant correlation.

The results of multivariate logistic regression analysis on the determinants of Mets, when factors other than the modified NCEP criteria were entered, demonstrated that HOMA-IR, as well as short duration of HD, BMI, and sex (men vs. women), were chosen as independent risk factors [Table 5].

## 4. Discussions

Mets is known as a cause of end-stage renal disease (ESRD) and CVD. It is reasonable to find high prevalence of Mets in HD patients [16,17]. However, the results are of interest due to the potentially conflicting nature of malnutrition and Mets in dialysis patients. In HD patients, whether Mets-related risk factors depend on visceral adiposity or uremia per se remains unknown. Although the present study does not provide a clear answer for this, we demonstrated that Mets was more prevalent in HD patients as well as in non-dialysis general populations, despite the prevalence of patients with a BMI over than 25 being only 11.2%. Mets tends to become less prevalent with the duration of HD. In patients with Mets, however, the higher the degree of malnutrition developing, the greater the proportion of patients who have insulin resistance with inflammation. Therefore, abdominal obesity may also play an important role in atherosclerosis as well as malnutrition in HD patients. In contrast to the general population, obesity is associated with improved survival [18] and decreased hospitalization rate [18] among patients with ESRD. In addition, the association between obesity and improved prognosis remained significant even after adjustment for serum albumin [18]. It may be hypothesized that a higher level of adiposity may provide a survival advantage for patients with ESRD.

Regarding the report on the prevalence of Mets in HD patients, Young et al. showed that Mets is highly prevalent in incident dialysis [8]. However, the patients were studied at the initiation of dialysis therapy in contrast to our report dealing with maintenance HD patients. Moreover, fasting blood samples were not used for evaluating each metabolic component and BMI was used instead of WC. In this regard, our study is the first report showing the precise prevalence of Mets in maintenance HD patients.

There are accumulating data that (visceral) abdominal obesity and attendant risk factors are associated with increased risk for CVD [16,19]. In a prospective study (Quebec Cardiovascular Study) in which more than 2000 middle-aged men were followed over 5 years, two clinical characteristics associated with visceral obesity were the strongest independent risk factors for ischemic heart disease: fasting hyperinsulinemia and increased apolipoprotein B concentrations [20]. Abdominal obesity is often accompanied by insulin resistance and hyperinsulinemia [9]. This hyperinsulinemia may, in turn, contribute to increased CVD and stroke. Insulin resistance in HD patients has been reported to be an independent predictor of CVD and mortality [13]. In the present study, the distribution of HOMA-IR was similar to that report [13], which means that insulin resistance still remains after the initiation of RRT. However, it appears that the prevalence of insulin resistance becomes less with the duration of HD.

Serum albumin level itself was not a determinant of Mets in HD patients. However, there was a significant correlation between serum albumin levels and insulin resistance in patients with Mets, whereas the association was not seen in patients without Mets. Therefore, the significance of serum albumin is thought not to be a determinant of Mets, but rather an important component in the pathophysiology of Mets in HD patients. In the patients with Mets, hypoalbuminemia is associated with increased HOMA-IR. Comparing patients without Mets, the patients with Mets have significantly higher hsCRP levels. Therefore, in prevalent HD patients, insulin resistance may play an important role for atherosclerosis through the interaction between malnutrition and inflammation. In patients with Mets, the higher the degree of malnutrition developing, the greater the proportion of patients who have insulin resistance with inflammation. In this regard, it is reported that TNF-α could play a role in the development of insulin resistance in humans, both in muscle and in vascular tissue [21]. Sustained low-grade inflammation could be one factor that explains why CKD and CVD often develop simultaneously. It is well known that insulin resistance is associated with endothelial dysfunction [22], which underlies atherosclerotic CVD. HOMA-IR showed a negative correlation with HD duration in our study. However, because it was a weak correlation, the result should be interpreted with caution. Further study might be necessary to confirm the relationship between HOMA-IR and HD duration.

Mets has been exposed to vigorous critique [23], while others are arguing that Mets is of great value [24]. Moreover, a role of Mets remains unclear in maintenance HD patients. Our study may provide a clue to consider Mets as well as malnutrition and its related atherosclerosis through insulin resistance.

There are several limitations to the current study. The present study is a cross-sectional and observational study in a single hospital. However, we do not want to obtain any causality between Mets and cardiovascular events. Second, we evaluated Mets according to the modified NCEP criteria, because in Japan these criteria were authorized by the Japanese Society of Internal Medicine in 2005 by changing the definition of WC. Tanaka and Iseki et al. have already reported the relationship between the Mets and CKD [7] using these modified criteria. The relationship between NCEP criteria and modified criteria is well documented in their report. Indeed, the prevalence of Mets was 12.4% when NCEP criteria was used, while the prevalence increased up to 21.2% when modified criteria was used with a similar rate reported in the USA [6]. The discrepancy might be related to the difference in the prevalence and degree of obesity between the two countries [25]. Evaluation of nutritional status including prealbumin, muscle consumption, upper arm muscle circumference, and comprehensive score was not evaluated in this study. Therefore, full assessment of nutritional status was not performed. However, the objective of the present study was to reveal a relationship between malnutrition and atherosclerosis in terms of metabolic syndrome, which is known to be an independent risk factor for cardiovascular disorders. In order to discuss this issue, we focused on serum albumin levels being an important nutritional factor. It is no doubt that serum albumin, although affected by inflammation, plays an important role as one of many nutritional markers. Future study is necessary to clarify the association between nutritional status by precise nutritional assessment and Mets in patients undergoing HD. Finally, regarding a difference between % of males versus females in the present study, in an overview of regular dialysis treatment in Japan as of 31 December 2009 reported by the Japanese Society for Dialysis Therapy, there are, in Japan, 173,391 men versus 106,722 women in regular dialysis treatment, a ratio (Men/Women) of 1.72, which clearly shows a predominance of men over women, with a similarity to our study (men 97/women 44). Despite the limitations described above, we believe that the data obtained from this study provide evidence of an important issue considering nutritional status and abdominal obesity in maintenance HD patients.

In conclusion, we demonstrate that Mets is more prevalent in HD patients. Mets tends to become less prevalent with the duration of HD and the development of malnutrition. In Japanese HD patients, 100 cm^2^ of VFA corresponded to a WC of 85 cm in men and 90 cm in women, thus confirming the validity of the modified criteria. HOMA-IR and serum albumin were significantly correlated in HD patients with Mets, not in those without Mets. Malnutrition and insulin resistance may interact with metabolic syndrome in patients with prevalent HD.

## Figures and Tables

**Table 1:** Baseline characteristics of the patients.

	All	Men	Women	*P*
				(Men vs. Women)
N	141	97	44	
Age (years)	67 ± 11.8	68 ± 10.7	67 ± 12	0.974
HD vintage (months)	83 ± 69	75 ± 62	99 ± 79	0.053
Body height (cm)	161 ± 9.0	165 ± 6.9	153 ± 6.9	<0.001
Body weight (kg)	55.0 ± 10.9	58.2 ± 10.0	48.0 ± 9.4	<0.001
WC (cm)	83.9 ± 9.0	84.7 ± 8.7	82.3 ± 9.7	0.143
BMI (kg/m^2^)	21.1 ± 3.4	21.3 ± 3.2	20.5 ± 3.8	0.235
Current smoker, n	35	29	6	0.038
Diabetes mellitus (%)	49.6	55.7	36.4	0.034
TC (mg/dL)	152 ± 34	149 ± 33	162 ± 36	0.043
HDL-C (mg/dL)	47 ± 15	44 ± 14	51 ± 17	0.032
LDL-C (mg/dL)	76 ± 25	75 ± 24	78 ± 25	0.666
TG (mg/dL)	97 ± 69	94 ± 77	101 ± 47	0.585
FBS (mg/dL)	97 ± 33	100 ± 38	89 ± 21	0.072
Insulin (μU/mL)	7.3 ± 11.9	6.3 ± 5.3	9.5 ± 19.3	0.151
HOMA-IR	0.927 (0.593–1.906)	0.921 (0.590–1.991)	0.924 (0.593–1.772)	0.328
Serum albumin (g/dL)	3.67 ± 0.35	3.71 ± 0.33	3.60 ± 0.37	0.251
Systolic BP (mmHg)	143 ± 24	144 ± 25	142 ± 21	0.371
Diastolic BP (mmHg)	77 ± 13	77 ± 13	74 ± 14	0.104

Abbreviations: HD hemodialysis, WC waist circumference, BMI body mass index (kg/m^2^), TC total cholesterol, HDL-C high-density lipoprotein cholesterol, LDL-C low-density lipoprotein cholesterol, TG triglyceride, FBS fasting blood glucose, HOMA-IR Homeostasis Model Assessment of Insulin resistance, BP blood pressure.

**Table 2:** Prevalence of Mets component in HD patients.

	Men	Women
Waist ≥ 85 cm, n (%)	52 (53.6)	
Waist ≥ 90 cm, n (%)		11 (25.0)
HDL < 40 mg/dL, n (%)	41 (42.3)	13 (29.5)
TG ≥ 150 mg/dL, n (%)	11 (11.3)	7 (15.9)
FBS ≥ 110 mg/dL, n (%)	23 (23.7)	7 (15.9)
HTN ≥ 130/85 mmHg, n (%)	80 (82.5)	38 (86.4)

Abbreviations: Mets metabolic syndrome, HDL high-density lipoprotein, TG triglyceride, FBS fasting blood glucose, HTN hypertension.

**Table 3:** Characteristics of the patients with or without Mets.

	Mets+	Mets−	*p*-Value
Number of patients, n (%)	37 (26.2)	104 (73.8)	
Sex (Male/Female)	31:6	66:38	<0.05
Age (years)	67.3 ± 9.2	68.2 ± 11.9	NS
WC (cm)	92.3 ± 5.9	80.9 ± 8.1	<0.0001
HD duration (months)	54.5 ± 39.8	92.5 ± 73.8	<0.005
BMI (kg/m^2^)	23.4 ± 3.2	20.2 ± 3.1	<0.0001
Serum albumin (g/dL)	3.65 ± 0.37	3.68 ± 0.34	NS
HOMA-IR	1.818 (0.818–2.818)	0.788 (0.332–1.244)	<0.0001
hsCRP (mg/dL)	0.208 (0.001–0.700)	0.083 (0.001–0.245)	<0.05
TC (mg/dL)	151 ± 37	153 ± 33	NS
HDL-C (mg/dL)	37 ± 10	49 ± 15	NS
LDL-C (mg/dL)	79 ± 27	75 ± 24	NS
TG (mg/dL)	136 ± 109	83 ± 39	0.0001
FBS (mg/dL)	109 ± 14	93 ± 27	0.005
Systolic BP (mmHg)	148 ± 23	143 ± 24	NS
Diastolic BP (mmHg)	75 ± 14	77 ± 13	NS

Abbreviations: WC waist circumference, HD hemodialysis, BMI body mass index, HOMA-IR Homeostasis Model Assessment of Insulin resistance, hsCRP high-sensitive C-reactive protein, TC total cholesterol, HDL-C high-density lipoprotein cholesterol, LDL-C low-density lipoprotein cholesterol, TG triglyceride, FBS fasting blood glucose, BP blood pressure.

**Table 4:** Univariate regression analysis associated with HOMA-IR.

	R	*p*-Value
Age (years)	0.150	0.414
HD duration (months)	0.120	0.513
Serum albumin (g/dL)	−0.401	0.023
hsCRP (mg/dL)	0.121	0.509
WC (cm)	0.077	0.678
BMI (kg/m^2^)	0.046	0.803
TC (mg/dL)	0.158	0.393
LDL-C (mg/dL)	0.208	0.393
TG (mg/dL)	0.056	0.756
Systolic BP (mmHg)	−0.315	0.078
Diastolic BP (mmHg)	−0.333	0.063

Abbreviations: HOMA-IR Homeostasis Model Assessment of Insulin Resistance, HD hemodialysis, hsCRP high-sensitive C-reactive protein, WC waist circumference, BMI body mass index, LDL-C low-density lipoprotein cholesterol, TG triglyceride, BP blood pressure.

**Table 5:** Univariate and multivariate logistic regression analysis on the determinants of Mets, when factors other than the modified NCEP criteria were entered.

	Multivariate		Univariate	
	OR (95% CI)	*p*-Value	OR (95% CI)	*p*-Value
Sex, vs. women	7.226 (1.618–32.264)	<0.01	2.975 (1.138–7.777)	<0.01
Age (years)	0.980 (0.930–1.033)	NS	0.992 (0.966–1.026)	NS
BMI (kg/m^2^)	1.344 (1.130–1.598)	<0.001	1.362 (1.187–1.562)	<0.0001
LDL-C (mg/dL)	1.001 (0.981–1.021)	NS	1.007 (0.992–1.022)	NS
Serum albumin (g/dL)	0.352 (0.042–2.983)	NS	0.806 (0.272–2.384)	NS
HD duration (months)	0.986 (0.976–0.997)	<0.05	0.989 (0.981–0.997)	<0.01
log HOMA-IR	5.230 (1.286–21.280)	<0.05	6.164 (2.025–18.759)	<0.01
log hsCRP	1.250 (0.526–2.970)	NS	2.109 (1.103–4.034)	<0.05

Abbreviations: BMI body mass index, LDL-C low-density lipoprotein cholesterol, HD hemodialysis, HOMA-IR Homeostasis Model Assessment of Insulin Resistance, hsCRP high-sensitive C-reactive protein.

## Data Availability

The datasets used and/or analyzed during the current study are available from the corresponding author on reasonable request.
